# The relationship between financial decisions and equity risk

**DOI:** 10.1016/j.heliyon.2022.e10036

**Published:** 2022-07-31

**Authors:** Anh Thi Lan Nguyen, Duy Van Nguyen, Nam Hoang Nguyen

**Affiliations:** a Faculty of Economics, Tay Bac Univeristy, Viet Nam; b Faculty of Finance and Banking, Dai Nam University, Viet Nam

**Keywords:** Financial decisions, Equity risk, Funding decision, Investment decision, Working capital decision

## Abstract

This study was conducted to determine the impact of financial decisions on equity risk in enterprises. The factors representing financial decisions include investment decisions, working capital decisions, and funding decisions. The equity risk is represented by beta in firms. Research and analysis of panel data with generalized least squares (GLS) via industry construction companies listed on the Vietnam Stock Exchange from 2015 to 2019. The data analysis results show that investment decisions do not affect equity risk, working capital decisions have a positive impact on equity risk, and funding decisions have a negative impact on equity risk. Furthermore, this study shows that agency theory does not exist in the relationship between financial decisions and equity risk in industry-construction enterprises in Vietnam. From this result, the authors also provide implications to help investors and corporation managers make decisions according to their goals based on signs of financial decisions.

## 1 Introduction

For listed companies, investors always pay special attention to the volatility of stocks on the market [1, 2]. Different investors have different perspectives and risk exposures when making investment decisions [3]. In particular, some investors prefer stocks or groups of industries with a high level of risk to expect greater returns. At the same time, some investors want low returns with a medium level of risk [3]. The criterion to assess the level of risk for each stock on the market is described by the beta or equity risk index. This beta index indicates whether a portfolio of stocks has a high or low level of risk compared to the market. Therefore, this is also an essential criterion in the investment decisions of investors in the market.

The financial decisions of management in the enterprise aim to bring more profit and avoid controllable risks [4, 5, 6]. Financial decisions can be suitable regarding expected profits but can also bring about failures [4, 5, 6]. But failure will bring risks to businesses. It is often due to several reasons: wasting capital, over-investment or under-investment, and poor cash flow planning makes capital turnover worse [7, 8, 9]. However, the cause that companies encounter often comes from a poor financial management strategy.

Many studies have been conducted to assess the impact of financial decisions on equity risk. Most studies show a close relationship between financial decisions such as the decision on capital source, investment capital, dividend payout ratio on equity risk [10, 11, 12]. Besides that, Zopounidis and Doumpos [13] used the factor "Multi-criteria decision support" to help make financial decisions by assessing aspects such as company operations, investments, financial issues, and credit; The authors have pointed out the advantages of this technique in financial decision making [13].

This study focuses mainly on agency theory to explain the relationship between financial decisions and equity risk [5, 6, 14]. The different benefits between CEO and business ownership or shareholders will impact financial decisions on equity risk in different economic environments and other types of companies. Companies whose financial decisions affect equity risk, in the long run, are of great interest to businesses in the industry, construction, and real estate investments. Because the asset structure is mainly in long-term assets when the facilities items account for a large proportion of capital. At the same time, the investment items of enterprises in this industry have strategies for long-term development. Therefore, financial decisions in this field always need risk assessments to control risk in general and equity risk in particular. Furthermore, in Vietnam, the construction and innovation process is taking place strongly. Hence, industry construction companies need to have good financial management, and financial decisions need to be made smart to equity risk control.

Although there has been some research on investment decisions, equity risk is still limited. At the same time, many studies have mixed opinions between financial decisions and equity risk [15, 16, 17, 18]. In addition, different economic markets can yield different results between financial decisions and equity risk. Moreover, there are no specific studies specifically within the scope of research in Vietnam. Therefore, this study was conducted to determine the impact of financial decisions on equity risk. The research will help determine the financial decisions affecting the equity risk of enterprises so that managers and investors can make decisions that are in line with the goals.

## 2 Literature review

### 2.1 Agency theory, financial decisions, and equity risk

Agency theory, in this case, will show that the behaviors in the ownership and executive roles are different [19, 20, 21]. The nature of the relationship between the contracts with the stakeholder in an enterprise (for example, managers and shareholders). Agency theory is also referred to as manager behavior, organizational behavior, or strategic theory in the enterprise [22, 23]. Individuals who own shares will make decisions that benefit the company and shareholders rather than personal interests. CEOs without ownership will make decisions with different risks and private benefits [19, 20, 24, 25, 26, 27]. Managers can make financial decisions that benefit businesses or individuals without regard to the interests of shareholders [20]. Therefore, in this situation, the management's financial decisions may increase the risk for shareholders. Therefore, to control risky behavior for shareholders, agency costs are incurred to monitor the CEO's activities. When this agency cost is effective, it will bring positive benefits or reduce equity risk to shareholders while still bringing benefits to the corporation and shareholders as expected.

### 2.2 Financial decision

Financial decisions of enterprises are aimed at achieving the desired profits of the business in both the short and long term [4, 5, 28, 29, 30]. Managers make financial decisions based on technical analysis or market information [31]. To create more accurate decisions, individuals must regularly study and update their knowledge and market knowledge to avoid information errors or delay in information compared to others with ongoing financial markets [32, 33]. In addition to technical analysis, psychological and emotional factors also influence the decisions of individuals [34, 35, 36].

The financial strategy comprises three interconnected decisions: investment, funding, and working capital [21]. If the goal of a company is to maximize profits, it must find the best combination of the three types of financial decisions. According to Mallette [37], an organization's economic strategy is vital to the company it must be assessed and altered regularly, just like the operational plan [37]. The study also claims that the financial system's evaluation must align with the company's operations, needs, and specificities. As a result, the description of economic methods used by enterprises is a topic that has gotten increased attention. For example, Valenciano et al. [38] published research on financial procedures in enterprises, taking into account the features of the organization [38]. They discovered that most enterprises set an optimal leverage ratio, use investment evaluation techniques, have traditional management based on budgets and returns on investments, and rarely use methods like Economic Value Added or Balanced Scorecard or financial ratios to analyze profitability.

#### 2.2.1 Investment decision

Investment is the allocation of capital to bring about expected business performance [39]. At the same time, money, material, and human resources are also used in the investment. And investment decisions are based on an analysis of the project's feasibility to reduce the risks encountered [40]. Therefore, the investment decision is considered an important decision in determining the performance of enterprises [16]. Investment items in money or facilities people are all aspects that reflect the investment decisions of the business. At the same time, the most obvious is the factor related to the assets that the company invests in [16]. At the same time, two types of assets are shown to be related to fixed assets and current assets of the business [16].

#### 2.2.2 Funding decision

Funding decision identifies and uses funding sources characterized by long-term debt and short-term debt for investments [16]. In addition, funding sources can come from both internal and external sources. Internal funding can be obtained from retained earnings, while external funding sources involve loans or corporate share issuance for listed companies [5, 16]. The balance of capital use plays a vital role in improving operational efficiency and reducing associated risks. For example, the more leverage or debt used, the more profits will be increased in good business performance. On the contrary, if the use of borrowed capital is high, the performance is poor (ROE is lower than the borrowing rate), the more loans will make the company more difficult.

#### 2.2.3 Working-capital decisions

Managing short-term assets and liabilities in a way that assures the adequacy of resources for corporate operations is a part of working capital choices. The short-term assets or liabilities are considered while making working capital choices [17]. Working capital policies attempt to manage current assets (usually cash and cash equivalents, inventories, and debtors) as well as short-term borrowing to achieve good cash flows and returns.

### 2.3 Equity risk

The risk management function affects the cost of equity [18]. Equity risk is associated with the gap between the actual return and the expected return for shareholders [18]. The greater the disparity, the greater the danger [41]. The beta coefficient is a widely used metric for assessing the risk of investing in stocks. A company's beta assessment or estimation is critical for capital expenditure, performance, and extraordinary profits. Because a greater beta suggests a higher cost of equity for a company, beta can be a helpful way to gauge its equity risk [18, 41, 42]. When a company's cost of equity exceeds expectations or investors' expected returns fall short of expectations, the stock price suffers.

According to the CAPM model, market risk is assessed by market beta, the amount of risk that a single investment contributes to a portfolio of all traded assets. A stock's beta is a measure of a stockholder's projected return compared to the market. Thus, the only difference in predicted returns between the two investments is their betas [43]. Because equity risk has two components: firm-specific risk (or unsystematic risk) and market risk, Fairchild [44] and Crowther & Seifi [45] advocate for the usage of beta (or systematic risk). Specific risk is a metric that analyzes price changes in stocks caused by business-specific factors, and it is used to calculate the risk of a company's stock. Specific risk is a metric that evaluates changes in stock prices that are unrelated to market volatility and occur for business-specific reasons.

On the other hand, market risk assesses how sensitive a company's stock price is to market swings. As a result, business-specific hazards can be minimized for investors who buy a broad portfolio of equities. However, this is not practicable in the case of market risk. Even if a corporation has a well-diversified portfolio, the risks that influence most assets in the market will continue [44]. When the systematic risk is removed, an individual stock's risk is measured by its relative volatility to the market rather than its standard deviation [45]. Assuming adequate diversification, equity risk can be classified as market risk. Businesses with more volatile stocks than the market are riskier than those with less volatile stocks.

## 3 Method

### 3.1 Research model

The research model is mainly referenced from [15, 46]. At the same time, in addition to the main independent variables for financial decisions, the research model also uses the control variable SIZE and the sale growth variable. The model is presented:

$$Equtiyr\;isk_{it}=\alpha_{i}+\beta_{1}Financial\;decisions_{it}+\beta_{2}Control\;variables_{it}+\varepsilon_{it}$$

Details of the variables are described in Table 1.

**Table 1:** Variables description.

Variables	Symbol	Definition
***Dependent variable***:		
Equity risk	Beta	Beta of share i = (Covariance of stock i with market category)/(Variance of the market portfolio)
***Independent variables***:		
Investment decision	INV	*I*_*i,t*_*/K*_*i,t-1*_*; I:* represents the net investment of firm I during the period t; K: represents the lagged Net Fixed Assets
Funding decision	SL	Short-term liability/Total asset
	LL	Long-term liability/Total asset
Working capital decisions	CCC	The cash conversion cycle
***Control variables***		
Firm size	SIZE	Ln (total assets)
Growth of revenue	GROWTH	(Revenue_t_ – Revenue_t-1_)/Revenue_t-1_

#### 3.1.1 Dependent variables

**BETA:** This is the variable that represents equity risk in companies. This index is a popular proxy for assessing the riskiness of a portfolio in the market [41]. This coefficient is measured based on the volatility of the stock code compared to the market. A beta greater than 1 indicates a high level of risk.

#### 3.1.2 Independent variables

**INV:** Investment decision in enterprises. This index is measured through the ratio of net investment in year t and fixed assets in the enterprise in year t-1 [15,15,46]. The higher the ratio, the greater the level of investment. A larger INV indicates higher volatility and increases equity risk [16].

**CCC:** Working capital decision in companies. This index is measured by the formula: AR + INV – AP. In which AR is The number of days accounts receivable; INV stands for the number of days inventories; AP stands for The number of days accounts payable. The larger the CCC, the higher the risk encountered in the company and the greater the equity risk [15, 17].

**SL, LL:** are two indicators of funding decisions in businesses. The use of borrowed capital or financial leverage is meaningful to expand the company easily, but it will also bring interest risk. When CEOs decide to use a lot of debt to maximize the tax shield and make the most of the external resources of the company. This will increase equity risk as a backdrop to ongoing company uncertainty [5, 16].

#### 3.1.3 Control variables

**SIZE:** firm size is used as the control variable. Companies scale up to expect more profit (cite). However, risks may be encountered when doing business in inefficient investment categories. In addition, the company expansion will cause some resources to be changed, leading to risk in management, making the business situation of companies also affected [15]. Therefore, SIZE will increase equity risk.

**GROWTH:** Revenue growth in the enterprise is also used as a control variable in the model. A good revenue growth rate will bring expected profit to the business and shareholders. Therefore, well-developed signals will interest investors, and shareholders' interests will be more assured [47]. Therefore, GROWTH will have a negative effect on equity risk.

### 3.2 Data collection

Data used in the study was collected from listed companies with 100 companies in industry construction from 2012 to 2019 on the stock exchange. Accessible channels include vietstock.vn, and FiinPro (We confirm that all information collected by companies is publicly available on this financial system). Since the period of this study is from 2020, data for 2020 as well as 2021 have not been collected. Therefore, this can be considered a limitation of this study. Nevertheless, the research data will be inputted into STATA16 software for analysis.

### 3.3 Data analysis

Data is collected with 100 companies in industry construction built from 2015 to 2019, so panel data analysis will be used in this study. The initial fixed effect (FEM) and random effect (REM) analysis models will be used. Hausman test is performed to find the model that fits the research data. The model will be reliable when there is no autocorrelation and heteroskedasticity. In case the model encounters the above phenomena, the GLS correction model will be used.

## 4 Results

Descriptive results show that the mean of BETA was 0.44, of which the largest is 1.48. The mean of INV was 3.75, and the largest was 39.27. The mean of CCC was 957.83 days; the maximum was 5280. The mean of LL was 0.33; the maximum was 0.76. The mean of SL is 0.16; the maximum is 0.59. Finally, the mean of total assets is 3.2 trillion, of which the largest is 16.4 trillion. Details are in Table 2.

**Table 2:** Descriptive.

Variable	Mean	Std. Dev.	Min	Max
BETA (ratio)	0.44551	0.473039	0	1.48
INV (times)	3.75722	9.47674	0	39.27951
CCC (days)	957.839	1410.63	-70	5280.5
LL (ratio)	0.33228	0.211687	0.03	0.76
SL (ratio)	0.16722	0.173029	0	0.59
ASSETS (trillion)	3.20	4.50	0.16	16.4
GROWTH (%)	4.975485	92.07981	-1.903503	2038.04
N = 500				

The regression analysis results with the effect of investment decision on equity risk show that INV does not affect equity risk (p-value greater than 0.05). This finding is supported by prior research finding no effect of investment decision on equity risk. Seeing companies' investment dec-isions in the construction industry is not a sign to help assess equity risk. However, the hypothesis is about the positive impact of investment decisions on equity risk. But with research data for specific industry construction, the investment items do not change the beta number. In other words, a business's investments do not directly affect equity risk. This study shows that whether companies have a high or low level of risk does not depend on investment decisions. However, the way capital is managed or used can be a direct source of volatility in equity risk. Details are in Table 3.

**Table 3:** Regression for Investment decision and equity risk.

	(1)	(2)	(3)	(4)
VARIABLES	OLS	FEM	REM	GLS
INV	0.00140	0.00537	0.00306	0.00140
	(0.00232)	(0.00382)	(0.00272)	(0.00231)
SIZE	0.0918∗∗∗	0.139∗∗∗	0.0947∗∗∗	0.0918∗∗∗
	(0.0145)	(0.0534)	(0.0210)	(0.0144)
GROWTH	-0.000121	-0.000388∗	-0.000312	-0.000121
	(0.000224)	(0.000200)	(0.000194)	(0.000223)
Constant	-2.118∗∗∗	-3.463∗∗	-2.214∗∗∗	-2.118∗∗∗
	(0.407)	(1.497)	(0.591)	(0.406)
Observations	463	463	463	463
R-squared	0.082	0.034		
Number of i		100	100	100

Standard errors in parentheses.

∗∗∗p < 0.01, ∗∗p < 0.05, ∗p < 0.1.

**Figure 1:**
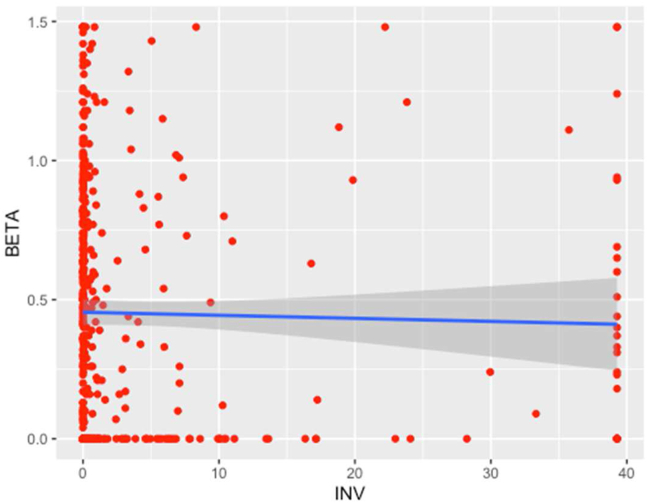
Relationship between investment decisions and equity risk.

The relationship between INV and Beta is depicted in Figure 1.

The working capital decision positively affects equity risk (positive beta and p-value less than 10%). It can be seen that the more days of capital turnover, the higher the equity risk. Today's results are similar to other studies that demonstrate a positive impact of the working-capital decision on equity risk [17]. The larger the CCC, the more affected cash flows with prolonged receivables. Or the large inventory makes inventory-related costs increase, affecting the business's business situation [17]. CCC is a signal for managers or investors to see whether the equity risk level of the company will tend to increase or decrease. Details are in Table 4.

**Table 4:** Regression for working capital decision and equity risk.

	(1)	(2)	(3)	(4)
VARIABLES	OLS	FEM	REM	GLS
CCC	2.83e-05∗	-1.26e-07	1.33e-05	2.83e-05∗
	(1.52e-05)	(1.94e-05)	(1.63e-05)	(1.51e-05)
SIZE	0.0878∗∗∗	0.140∗∗∗	0.0917∗∗∗	0.0878∗∗∗
	(0.0144)	(0.0528)	(0.0207)	(0.0143)
GROWTH	-0.000109	-0.000386∗	-0.000301	-0.000109
	(0.000222)	(0.000201)	(0.000194)	(0.000221)
Constant	-2.031∗∗∗	-3.454∗∗	-2.135∗∗∗	-2.031∗∗∗
	(0.401)	(1.478)	(0.579)	(0.399)
Observations	471	471	471	471
R-squared	0.092	0.030		
Number of i		100	100	100

Standard errors in parentheses.

∗∗∗p < 0.01, ∗∗p < 0.05, ∗p < 0.1.

**Figure 2:**
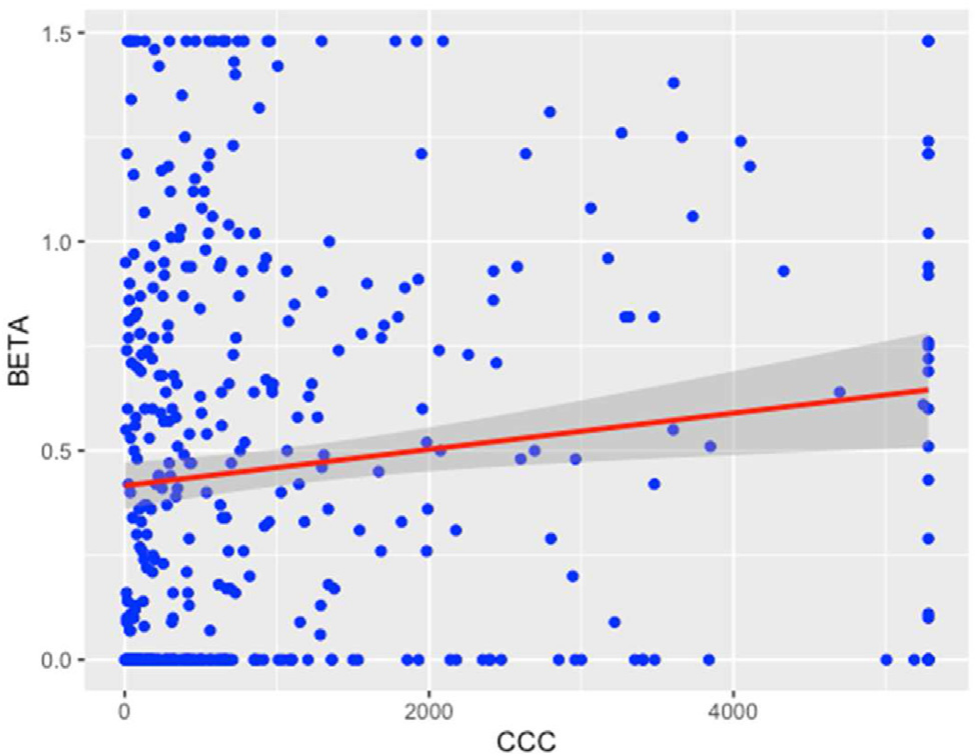
Relationship between working capital decision and equity risk.

The relationship between CCC and Beta is depicted in Figure 2.

Regarding the impact of funding decisions on equity risk, current and long-term liability ratios have opposite effects on equity risk (beta coefficients are both negative, and p-values are statistically significant at 5%). Using funding decisions reduces equity risk, suggesting that debt ratios reduce beta volatility. Decisions to increase the debt ratio bring more stability to the business. In a state of constant total assets, using debt makes the company less volatile than raising equity through equity financing. This result is more consistent with the trade-off theory than the pecking order theory. The activities of using debt or leverage play an essential role in maintaining stable operations of the business. Raising equity capital through the issuance of shares can be a bad signal that increases risks for the company [16]. Therefore, in the case of industrial enterprises - construction, loan capital will help reduce equity risk. Details are in Table 5.

**Table 5:** Regression for funding decisions and equity risk.

	(1)	(2)	(3)	(4)
VARIABLES	OLS	FEM	REM	GLS
LL	-0.212∗∗	-0.542∗∗	-0.271∗∗	-0.212∗∗
	(0.107)	(0.217)	(0.133)	(0.106)
SL	-0.570∗∗∗	-0.511∗	-0.544∗∗∗	-0.570∗∗∗
	(0.135)	(0.305)	(0.179)	(0.134)
SIZE	0.112∗∗∗	0.213∗∗∗	0.115∗∗∗	0.112∗∗∗
	(0.0146)	(0.0597)	(0.0212)	(0.0145)
GROWTH	-0.000144	-0.000414∗∗	-0.000333∗	-0.000144
	(0.000219)	(0.000199)	(0.000193)	(0.000218)
Constant	-2.502∗∗∗	-5.225∗∗∗	-2.587∗∗∗	-2.502∗∗∗
	(0.399)	(1.625)	(0.579)	(0.397)
Observations	471	471	471	471
R-squared	0.120	0.047		
Number of i		100	100	100

Standard errors in parentheses.

∗∗∗p < 0.01, ∗∗p < 0.05, ∗p < 0.1.

**Figure 3:**
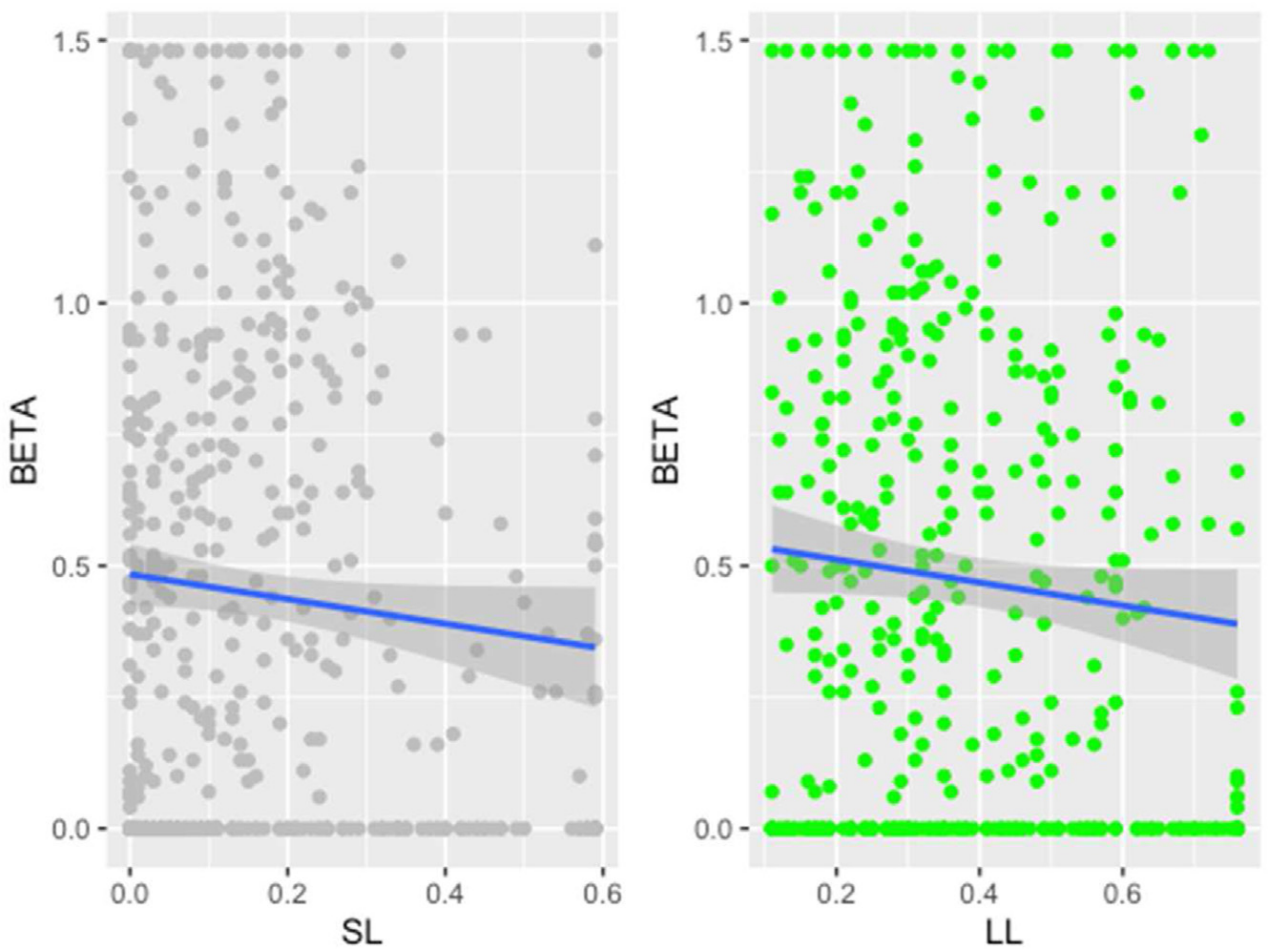
Relationship between funding decisions and equity risk.

The relationship between SL, LL and Beta is depicted in Figure 3.

Besides, the study also found a positive effect of firm size on equity risk (negative beta and significant p-value at 5%). Companies' firm size increase will crease equity risk. Increasing the size of the business makes it challenging to manage the breadth of the business. It can be seen that the management of business operations encounters specific problems when the company is large. Corporate stock prices can fluctuate more than the market when the business expands. At the same time, revenue growth also does not affect equity risk, showing that revenue is not a clear indicator to consider equity risk in construction industry businesses.

The study also compares the impact of financial decisions on equity risk by firm size. The results show that financial decisions are insignificant for smaller companies (the median with mean of assets is the cutoff between smaller and larger firms). Larger firms have the same financial decisions on equity risk (INV has no impact on equity risk, CCC has a positive effect on equity risk, and funding decisions have a negative impact on equity risk) (see Table 6). It can be seen that larger firms are more representative of research firms. On the other hand, smaller firms make financial decisions that are not yet decisive in changing equity risk. Details are in Table 6.

**Table 6:** The regression results are separated by firm size.

	Smaller firm size			Larger firm size		
BETA	(1)	(2)	(3)	(1)	(2)	(3)
INV	0.00229			0.00171		
	(0.00302)			(0.00367)		
CCC		1.41e-05			4.39e-05∗∗	
		(2.26e-05)			(2.00e-05)	
LL			-0.131			-0.395∗∗
			(0.141)			(0.161)
SL			-0.112			-1.009∗∗∗
SIZE	0.0802∗	0.0807∗	0.0866∗∗	0.127∗∗∗	0.119∗∗∗	0.146∗∗∗
	(0.0433)	(0.0427)	(0.0432)	(0.0237)	(0.0232)	(0.0227)
GROWTH	-0.000126	-0.000118	-0.000123	-0.0144	-0.0142	-0.0107
	(0.000227)	(0.000225)	(0.000225)	(0.0103)	(0.00996)	(0.00953)
			(0.196)			(0.183)
Constant	-1.786	-1.810	-1.900	-3.147∗∗∗	-2.959∗∗∗	-3.361∗∗∗
	(1.166)	(1.147)	(1.158)	(0.691)	(0.672)	(0.644)
Observations	222	230	230	241	241	241
Number of i	49	49	49	51	51	51

Standard errors in parentheses.

∗∗∗p < 0.01, ∗∗p < 0.05, ∗p < 0.1.

**Figure 4:**
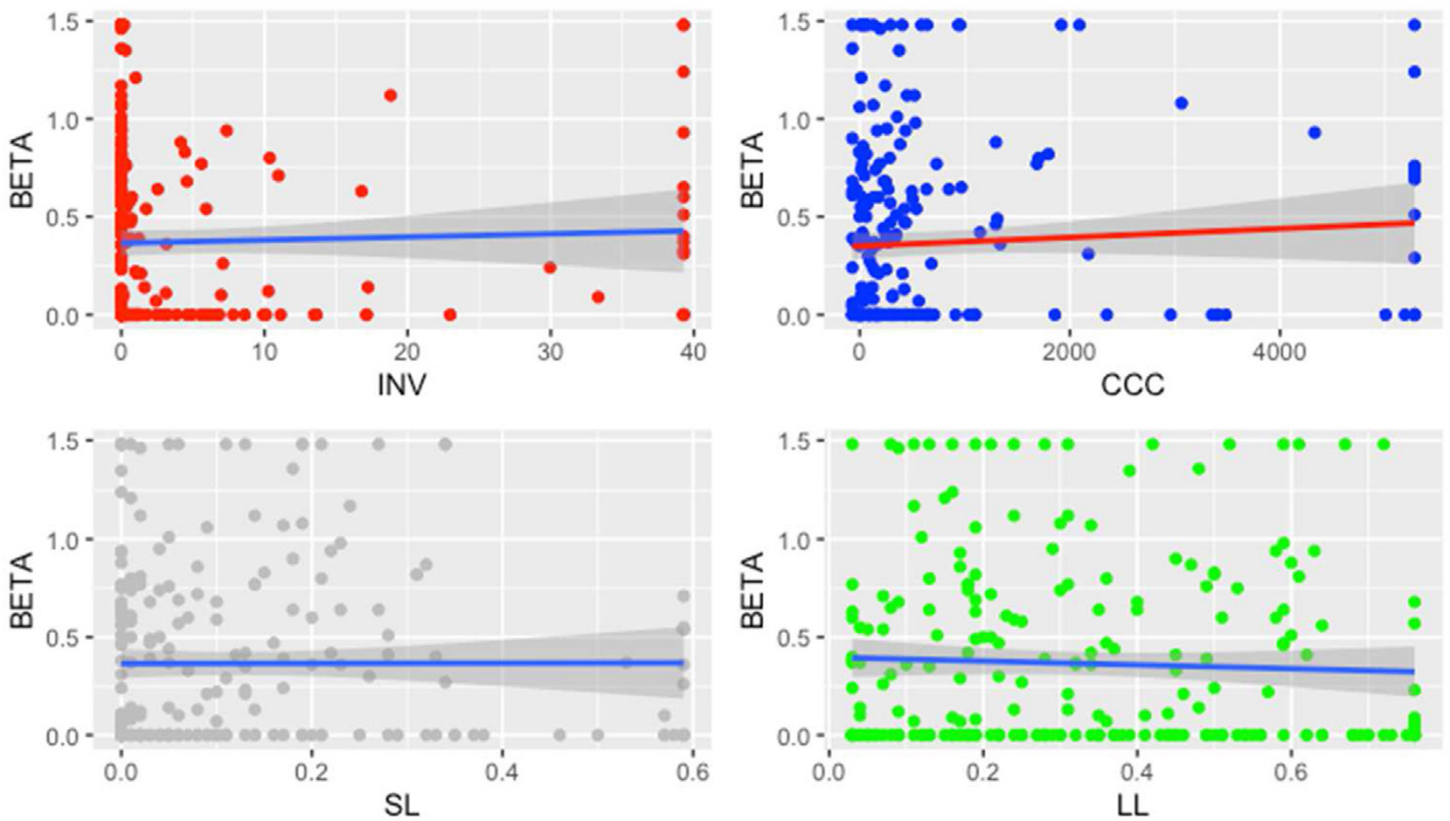
Regression for Smaller firm size.

The relationship between the financial decisions and Beta with Smaller firm size is depicted in Figure 4.

**Figure 5:**
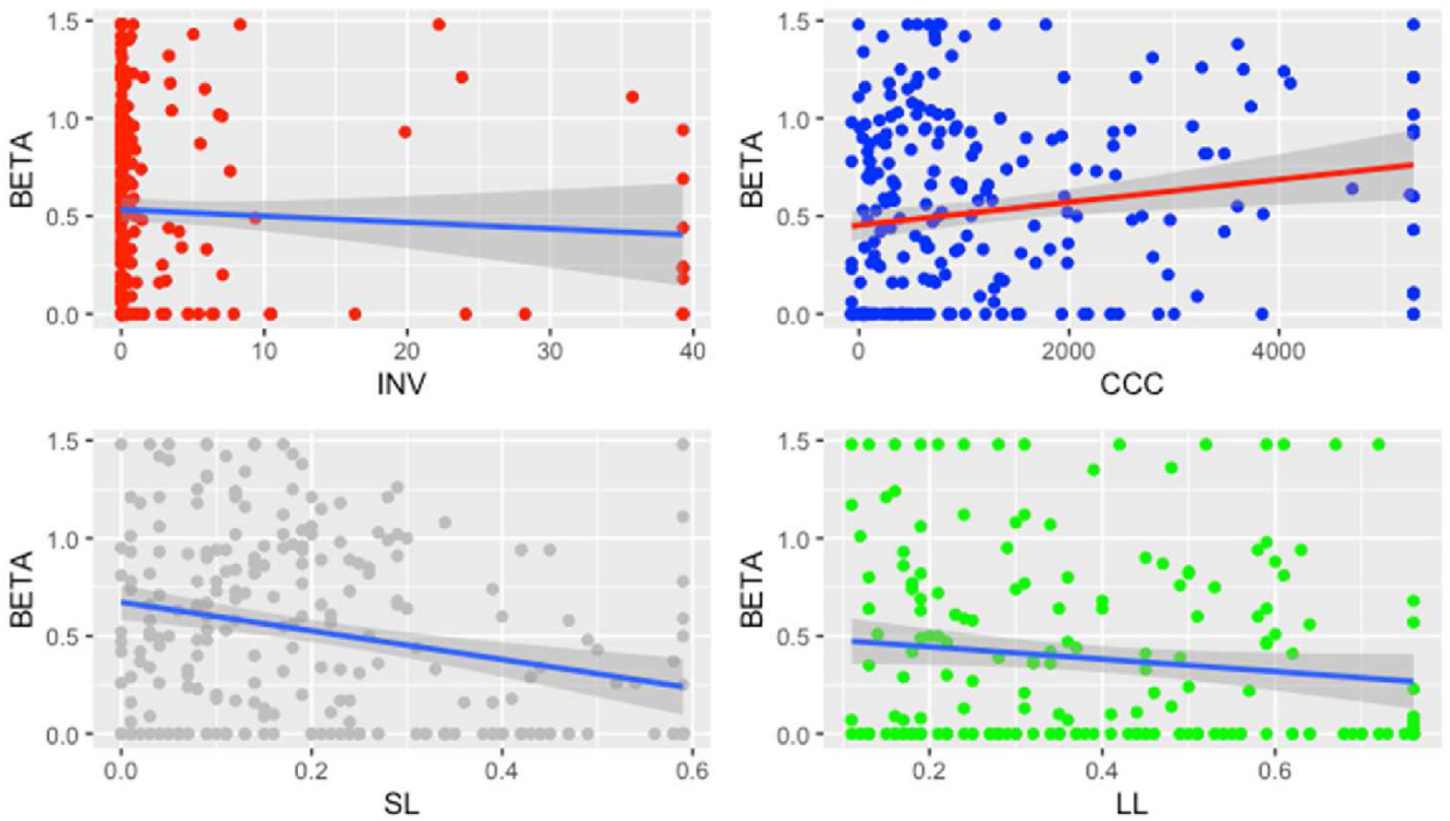
Regression for Larger firm size.

The relationship between the financial decisions and Beta with Larger firm size is depicted in Figure 5.

## 5 Conclusion and implications

### 5.1 Conclusion

The study has systematized the theoretical basis of financial decisions and equity risk in enterprises. The financial decisions are studied and described through three main components, including (1) Investment decision, (2) working capital decision, and (3) funding decisions. According to the pre-existing theories, these financial decisions can all bring risks to the companies. Also, in this study, equity risk is measured by the popular index beta, which is related to the volatility of a stock relative to the market. From the relevant theoretical literature, studies have found that the impact of the financial decision on equity risk is different in different contexts. Therefore, in this study, the authors examine this relationship in the context of enterprises in Vietnam.

Also, from previous studies, the authors have built a research model to assess the impact of financial decisions on equity risk. With data collected from 100 industry-construction enterprises listed on the Vietnam stock exchange. Using the GLS model for panel data analysis from 2015 to 2019. The analysis results show that the investment decision does not affect equity risk. Therefore, investment decisions are not meaningful in altering equity risk. The working capital decision has a positive impact on equity risk. Thus, attempts to reduce equity risk can be based on reducing working capital decisions and vice versa. Finally, funding decisions have a negative impact on equity risk in both current liability and long-term liability metrics. Therefore, reducing equity risk can increase funding decisions. From these research results, the authors also make some implications as follows.

### 5.2 Implications

**Theoretical implication:** The results of this study contribute to evidence on the impact of financial on equity risk in firms. Financial decisions such as working capital or liabilities bring about changes in equity risk in companies. The study will contribute to the research model of post-assessment related to financial decisions and equity risk. At the same time, the study also shows that the agency theory is not supported in this research context. With CCC having a positive effect on equity risk, CEOs want to reduce CCC to bring better firm performance results and an indicator of reducing equity risk for shareholders and future successors.

**Practical implication:** The working capital decision has a positive impact on equity risk. Therefore, the risk-loving investors with portfolios that can offer many returns but have the potential for high risk may consider higher CCC decisions. Businesses with high CCC will be an indication of high equity risk and when expect greater profits. The CCC index is an easy-to-follow index in the reports of companies, so this is an indicator to help investors be more proactive in their investment decisions. Besides, managers, depending on their strategic goals, adjust the CCC accordingly to control the equity risk of their company. Using both short-term and long-term liabilities capital structures reduces equity risk for funding decisions. Therefore, investors can also consider this a sign to make their investment decisions. Investors with a high-risk appetite may be interested in companies with lower debt ratios. Conversely, companies with low debt ratios are indicative of high equity risk. On the part of enterprise managers, using a lot of debt will reduce the equity risk.

### 5.3 Limitations and future research

**Limitations:** The study has shown the impact of financial decisions on equity risk through regression estimation of GLS panel data with 100 construction industry enterprises. However, the research still has certain limitations both subjectively and objectively: Firstly, the study to find out financial decisions on equity risk is limited to construction industry enterprises but has not yet been defined the general for companies, and classify each different industry group for comparison. Secondly, the study until the end of 2019 this period has not been affected much by COVID-19. However, from 2020, COVID-19 will severely affect companies in general. Finally, the study has not yet estimated how this factor changes the influence of financial decisions on equity risk. Third, the study only stopped analyzing the GLS correction model without considering the endogenous phenomenon using GMM or 2SLS for correction.

**Future research:** Given the above limitations, the authors suggest that future research can fill these gaps or limitations. Firstly, further studies need to collect data for all industries to provide a general assessment problem for enterprises in Vietnam. At the same time, the goal of compare how differences between sectors are also made more accessible. Second, further research can further estimate the covid-19 pandemic to measure the impact of this pandemic on activities within companies. Finally, the following studies may consider more endogeneity and use more complex models such as GMM and 2SLS.

## Declarations

### Author contribution statement

Anh Thi Lan Nguyen: Conceived and designed the experiments; Performed the experiments; Analyzed and interpreted the data; Wrote the paper.

Duy Van Nguyen: Performed the experiments; Analyzed and interpreted the data; Contributed reagents, materials, analysis tools or data; Wrote the paper.

Nam Hoang Nguyen: Analyzed and interpreted the data; Wrote the paper.

### Funding statement

This research did not receive any specific grant from funding agencies in the public, commercial, or not-for-profit sectors.

### Data availability statement

Data will be made available on request.

### Declaration of interests statement

The authors declare no conflict of interest.

### Additional information

No additional information is available for this paper.
